# Comparative phylotranscriptomics reveals glycolytic adaptation associated with prolonged larval development in the coastal slipper lobster *Crenarctus bicuspidatus*

**DOI:** 10.1080/19768354.2026.2643995

**Published:** 2026-03-21

**Authors:** Hyeongwoo Choi, Hyeongju Choi, Chungkug Park, Won-Kyo Jung, Tae-Hoon Kim, Yun Keun An

**Affiliations:** a Department of Earth Systems and Environmental Sciences, Chonnam National University, Gwangju, Korea; b Department of Environmental Oceanography, Chonnam National University, Yeosu, Korea; c Jeollanam-do Institute of Ocean & Fisheries Science Future & Fisheries Research Institution, Yeosu, Korea; d Research Center for Marine-Integrated Biomedical Technology, Pukyong National University, Busan, Korea; e Department of Aquaculture, Chonnam National University, Yeosu, Korea

**Keywords:** Comparative transcriptomics, gene family dynamics, glycolytic adaptation, positive selection, scyllaridae

## Abstract

The molecular basis of ecological adaptation in marine crustaceans remains poorly understood, particularly in non-model species such as the coastal slipper lobster (*Crenarctus bicuspidatus*). This study presents the first comprehensive analysis of the mitochondrial genome and transcriptome of *C. bicuspidatus* using high-throughput sequencing. Comparative transcriptomic analysis across 13 crustacean species identified 250 single-copy orthogroups. Gene family evolution analysis revealed 28 gene family expansions and 152 contractions in *C. bicuspidatus*. KEGG enrichment analysis of the expanded gene families showed strong representation of energy-related pathways, particularly pyruvate metabolism. Furthermore, positive selection analysis identified several genes involved in pyruvate metabolism and the citrate cycle (TCA cycle), including specific amino acid substitutions in the pyruvate dehydrogenase complex, a key enzyme in energy conversion. These findings suggest that *C. bicuspidatus* has undergone coordinated genomic adaptations to enhance energy metabolism, likely to support the high energetic demands of its extended larval development. This study highlights glycolytic adaptation as a key driver of life-history diversification in slipper lobsters.

## 1. Introduction

Marine crustaceans display remarkable ecological and morphological diversity, inhabiting a wide range of environments from deep-sea habitats to dynamic coastal ecosystems. Despite their ecological importance and evolutionary significance, the molecular mechanisms driving their diversification remain poorly understood, particularly in non-model taxa such as slipper lobsters (family: Scyllaridae). Within this group, *C. bicuspidatus* represents a unique lineage characterized by an exceptionally prolonged larval development period, which distinguishes it from many other crustaceans. However, its phylogenetic position remains unresolved, and the genomic adaptations supporting such a distinct life-history strategy – particularly those required for sustained energy metabolism during its extended larval phase – have yet to be explored.

Mitochondrial and nuclear markers have long been used to infer phylogenetic relationships among crustaceans (Eyun et al. 2017). However, relying on a limited number of genes can result in incomplete or biased phylogenetic reconstructions, particularly in lineages that have experienced rapid or convergent evolution (Choi et al. 2025). Recent advances in high-throughput transcriptome sequencing now enable genome-scale phylogenetic inference by providing access to hundreds of orthologous protein-coding genes without the need for a complete reference genome. This approach not only enhances phylogenetic resolution but also yields functionally informative data that can elucidate the molecular basis of ecological adaptation (Cheon et al. 2020). Transcriptome-based analyses have proven especially useful in resolving complex evolutionary histories in non-model marine organisms, as demonstrated in recent studies (Libourel et al. 2023).

In this study, we present the first integrated mitochondrial and transcriptomic dataset for *C. bicuspidatus* to investigate its phylogenetic placement and molecular signatures of adaptation. Through ortholog-based comparative analyses across 13 crustacean species, we examine gene family evolution, identify positively selected genes, and explore the functional implications of metabolic pathway enrichment. Notably, the use of transcriptomic data enables comprehensive genomic analyses in the absence of a reference genome, allowing for the identification of coding sequences, orthologous genes, and adaptive signatures across species. This highlights not only the utility of transcriptome-based approaches in non-model organisms such as *C. bicuspidatus*, but also underscores the growing potential of transcriptomics to inform phylogenetic reconstruction and adaptive evolution in diverse crustacean lineages.

## 2. Materials and methods

### 2.1. Sample collection and sequencing

Healthy specimens of *Crenarctus bicuspidatus* were collected from the coast of Yokji Island (34°43′33′′ N, 128°14′45′′ E), South Korea, at a depth of 30–40 m in June 2024 using pot nets (36.5 cm in diameter, 70 cm in height, and 2 cm stretched mesh size). A single pot net was deployed and left submerged for 24 h before retrieval. Upon collection, individuals were dissected in the laboratory. Two muscle tissue samples from a single individual were immediately frozen in liquid nitrogen and stored at –80 °C.

Genomic DNA was extracted using the QIAGEN Blood & Cell Culture DNA Mini Kit (QIAGEN, Hilden, Germany), following the manufacturer’s instructions. DNA libraries were then prepared using the TruSeq DNA Nano 550 Kit (Illumina, San Diego, CA, USA). Total RNA from muscle tissue was extracted using the RNeasy Mini Kit (QIAGEN, Hilden, Germany) following the manufacturer’s protocol, and all procedures were conducted by JS Link (JS Link Inc., Seoul, Republic of Korea). Muscle tissues were placed in liquid nitrogen–pre-chilled microcentrifuge tubes and homogenized using a bead beater at 50 Hz for 10 s, repeated three times. Homogenized samples were mixed with 350 µl of Buffer RLT and thoroughly vortexed to lyse the tissue. The lysates were centrifuged at maximum speed for 3 min, and the cleared supernatants were transferred to new tubes without disturbing the debris pellet. An equal volume of 70% ethanol was added to each lysate, and the mixture was applied to a RNeasy Mini spin column. Columns were centrifuged and subsequently washed with Buffer RW1 followed by two washes with Buffer RPE according to the kit instructions. RNA was eluted by applying 50 µl of RNase-free water directly onto the membrane and centrifuging for 1 min at 8000 × g. RNA purity was measured using a NanoDrop 8000 spectrophotometer (Thermo Fisher Scientific, Waltham, MA, USA), and RNA integrity was assessed with a Bioanalyzer 2100 (Agilent Technologies, Santa Clara, CA, USA) to determine the RNA Integrity Number (RIN). mRNA sequencing libraries were prepared according to the manufacturer’s instructions for the TruSeq Stranded mRNA Library Prep kit (Illumina, San Diego, California, USA).

Both DNA and RNA libraries were sequenced on the NovaSeq 6000 platform (Illumina) to generate raw sequencing data. Prior to assembly, low-quality reads and those containing ambiguous bases (Ns) were filtered out using Trim_Galore (v0.6.10) (Martin 2011), yielding clean reads for downstream analysis.

### 2.2. Mitochondrial genome and transcriptome assembly

The mitochondrial genome of *C. bicuspidatus* was assembled *de novo* using MITOZ (v3.6) (Meng et al. 2019) with clean reads. Annotation and gene verification were performed using both MITOS1 and MITOS2 (Bernt et al. 2013). The circular mitochondrial genome was visualized using Circos (v0.69-8) (Krzywinski et al. 2009).

*De novo* transcriptome assembly was conducted using Trinity (v2.8.5) (Haas et al. 2013). Coding regions within the assembled transcripts were predicted using TransDecoder (https://github.com/TransDecoder/TransDecoder). To reduce redundancy and retain the longest transcript isoforms, the sequences were clustered using CD-HIT EST (v4.8.1) (Fu et al. 2012).

### 2.3. Phylogenetic analysis, and divergence time estimation

A species tree for the family Scyllaridae was reconstructed using 13 mitochondrial protein-coding genes (PCGs) from ten species, with *Panulirus ornatus* used as the outgroup. A total of nine complete mitochondrial genomes were downloaded from NCBI. Each PCG was aligned separately using MAFFT (v7.505) (Katoh et al. 2002), after which the alignments were concatenated. The best-fit substitution models for each gene were selected using PartitionFinder2 (v2.1.1) (Lanfear et al. 2016) based on the Bayesian Information Criterion (BIC). Phylogenetic trees were constructed using both maximum likelihood (ML) and Bayesian inference (BI) methods. For ML analysis, RAxML-NG (v1.1.0) (Kozlov et al. 2019) was used with 1,000 bootstrap replications to assess support. BI analysis was conducted using MrBayes (v3.2.6) (Ronquist et al. 2012), with four independent Markov Chain Monte Carlo (MCMC) runs of 1 × 10^6^ generations each. Trees were sampled every 1,000 generations, with the first 25% discarded as burn-in. The resulting ML tree was visualized using FigTree (v1.4.4) (http://tree.bio.ed.ac.uk/software/figtree).

In selecting taxon sampling, we initially aimed to reconstruct the order-level placement of *C. bicuspidatus* within Decapoda. However, due to the limited availability of high-quality decapod transcriptomes in public repositories, we broadened the sampling to 13 crustacean species spanning Malacostraca, Thecostraca, Copepoda, Branchiopoda and Hexapoda. This approach enabled the construction of a robust single copy ortholog matrix for phylogenomic inference. Single copy orthologous genes among 13 crustacean species were identified using OrthoFinder2 (Emms and Kelly 2019). The analysis included eight Malacostraca, one Thecostraca, two Copepoda, one Branchiopoda, and one Hexapoda species. Only the longest isoform per gene was used. DIAMOND (v2.0.15) (Buchfink et al. 2015) was employed to perform fast sequence similarity searches for ortholog detection.

Divergence time estimation was conducted using MCMCtree in PAML based on 250 single-copy orthologous coding sequences (total alignment length: 111,165 bp). An independent rate model was specified by setting ‘clock = 2’. The species tree inferred by OrthoFinder2 was used as the input. Four calibration points from the TimeTree database (Kumar et al. 2017) were applied: *Homarus americanus*–*Penaeus chinensis* (161–447.2 million years ago [MYA]), *Portunus trituberculatus*–*Hyalella Azteca* (169–388.2 MYA), *Amphibalanus amphitrite*–*Eurytemora affinis* (101.1–540.6 MYA), and the root age (275–541 MYA).

### 2.4. Gene family expansion, positive selection analysis, and functional annotation

Significantly expanded or contracted gene families were identified using the Computational Analysis of gene Family Evolution tool (CAFE, ver. 5.0.0) (Mendes et al. 2020), based on the species tree inferred above. Orthologous gene families were filtered using the following criteria: (1) families absent in five species, and (2) any family with more than 150 gene copies in a single species (Choi et al. 2025). Statistically significant expansions or contractions were determined using Viterbi *p*-values (*p* < 0.05). Additionally, a Z-test (Z > 1.96) was applied to identify gene families uniquely enriched in *C. bicuspidatus* (Choi et al. 2025).

To identify positively selected genes (PSGs), amino acid sequences were aligned using MAFFT. The corresponding 250 single-copy coding sequences were then aligned at the codon level using PAL2NAL (ver. 14) (Suyama et al. 2006). Rapidly evolving genes were identified using the branch-site model implemented in *codeml* from PAML (ver. 4.10.5) (Yang 2007). Null and alternative models were compared, with all parameters held constant except for ‘fix_omega.’ Each model was run across all 250 datasets. Likelihood ratio tests (LRTs) were conducted, and chi-square (χ^2^) distributions were used to evaluate model fit. Genes were identified as PSGs if the LRT *p*-value was < 0.05 and the ω (dN/dS) ratio exceeded 1 (Choi et al. 2025). Positively selected residues were identified as amino acid substitutions unique to *C. bicuspidatus* and were further supported by high posterior probabilities using the Bayes Empirical Bayes (BEB) method. The three-dimensional structures of PSGs were predicted using the AlphaFold3 web server (https://alphafoldserver.com) and visualized using PyMOL (ver. 4.6) (Schrödinger and DeLano 2020).

The function of genes under positive selection and those that were significantly expanded were determined using eggNOG-mapper (ver. 2.1.12) (Cantalapiedra et al. 2021). Additionally, functional categories were identified using the Gene Ontology (GO) and Kyoto Encyclopedia of Genes and Genomes (KEGG) databases using KOBAS (Bu et al. 2021).

## 3. Results and discussion

### 3.1. General information on the mitochondrial genome and phylogenetic placement of *C. bicuspidatus*

A total of 322,893,254 (48.76 Gb) and 57,434,074 (5.8 Gb) raw reads were generated for the genome and transcriptome of *C. bicuspidatus*, respectively, using the Illumina platform. These clean reads were used to assemble the mitochondrial genome and transcriptome.

**Figure 1.**
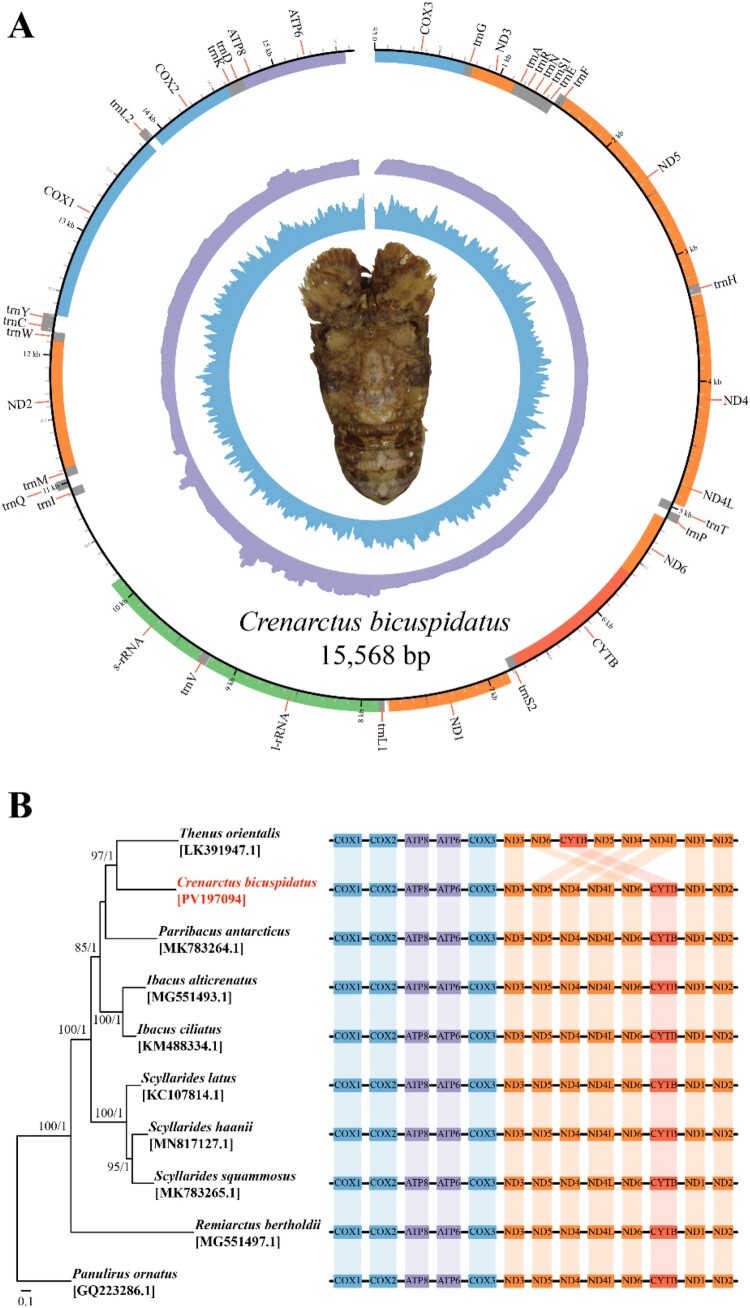
Mitochondrial genome information on *Crenarctus bicuspidatus*. (A) Circular map of the *C. bicuspidatus* mitogenome. The inner purple and blue rings indicate sequencing depth and GC content, respectively. The outermost ring shows gene annotations. (B) Maximum likelihood (ML) phylogenetic tree constructed using 13 protein-coding genes (PCGs) from 10 Scyllaridae species, with *Panulirus ornatus* used as the outgroup. Numbers at each node represent bootstrap values and Bayesian posterior probabilities, respectively. Mitochondrial gene order synteny maps are shown to the right of the tree, highlighting conserved and rearranged gene blocks across species.

The complete mitochondrial genome of *C. bicuspidatus* is 15,568 bp in length (Figure 1A; GenBank: PV197094), and includes all 13 protein-coding genes (PCGs), 22 transfer RNAs (tRNAs), and two ribosomal RNAs (rRNAs). This is the first complete mitogenome reported for *C. bicuspidatus*, contributing to the limited mitochondrial data currently available within the subfamily Scyllarinae, which previously included only two species: *Remiarctus bertholdii* (MG551497.1) and *Parribacus antarcticus* (MK783264.1) (Tan et al. 2019). Typically, closely related species group together in phylogenetic trees, reflecting their evolutionary proximity (Pavlopoulos et al. 2010). However, in the ML tree based on 13 PCGs, *C. bicuspidatus* (subfamily: Scyllarinae) unexpectedly clustered with *Thenus orientalis* (subfamily: Theninae) (Figure 1B). Notably, *R. bertholdii*, also a member of Scyllarinae, did not group with *C. bicuspidatus*. This unusual placement suggests that *C. bicuspidatus* may represent a distinct evolutionary lineage (Zheng and Lebeer 2020). Such a result could imply a taxonomic misclassification within the current framework or reflect convergent evolution that obscures true phylogenetic relationships (Gori et al. 2016; Balaban et al. 2019). Synteny alignment typically reveals conserved gene order among closely related species, supporting their evolutionary history. However, *T. orientalis*, which clusters with *C. bicuspidatus* in the ML tree, appears to have undergone syntenic rearrangements. This structural divergence further suggests that their apparent similarity in the phylogenetic tree may not accurately represent true evolutionary relatedness. These inconsistencies in syntenic patterns lend further support to the hypothesis of taxonomic misclassification or complex genomic rearrangements. To resolve these discrepancies and clarify evolutionary relationships within Scyllaridae, additional genomic and morphological analyses are needed. Furthermore, phylogenetic relationships can vary with both the number of taxa included and the amount of sequence data analyzed. To reduce this source of uncertainty, we reconstructed phylogenies using two independent datasets – 13 mitochondrial protein-coding genes and 250 transcriptome-derived single-copy orthologs. Despite this complementary approach, the limited availability of high-quality genomic and transcriptomic resources for Malacostraca remains a considerable challenge, as it restricts the inclusion of broader lineages that are necessary for resolving deeper evolutionary relationships. As additional mitochondrial, whole-genome, and transcriptome datasets become available, integrating these complementary data types will be essential for establishing a more comprehensive and well-resolved phylogenetic framework for Malacostraca.

### 3.2. Gene family dynamics and their functional implications

**Figure 2.**
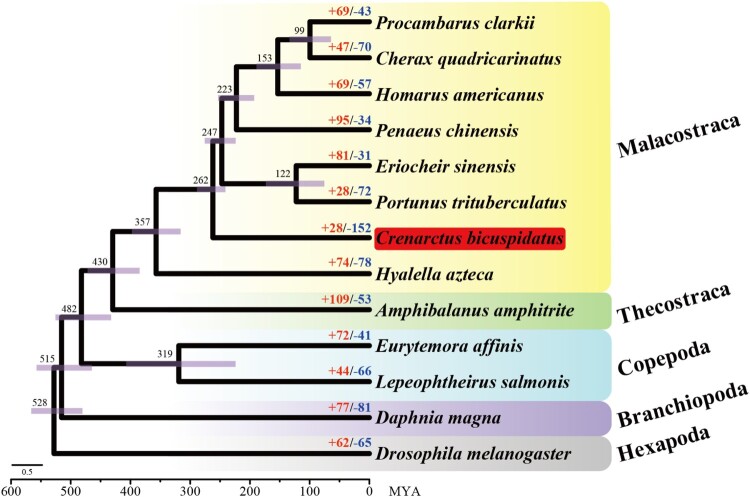
Phylogenetic reconstruction and gene family dynamics across 13 Crustacea species. ML tree constructed using 250 single-copy orthologs. Divergence times (in MYA) are shown at each node, with purple bars indicating 95% highest posterior density (HPD) intervals. Numbers at terminal nodes represent the number of significantly expanded (red) and contracted (blue) gene families in each lineage (*p* < 0.05). *Crenarctus bicuspidatus* is highlighted in red. Taxonomic groups are color-coded by clade: Malacostraca (yellow), Thecostraca (green), Copepoda (blue), Branchiopoda (purple), and Hexapoda (gray). The scale bar at the bottom indicates the number of substitutions per site.

Among the 250,870 protein-coding genes identified across 13 crustacean species, 217,500 (86.7%) were successfully assigned to orthogroups, indicating a high degree of shared genomic content across the taxa analyzed (Table 1). A total of 250 single-copy orthogroups were conserved across all species, providing a robust dataset for reconstructing phylogenetic relationships within Crustacea. Using 250 high-confidence single-copy orthologous genes, we reconstructed a time-calibrated phylogenetic tree (Figure 2). In this analysis, *C. bicuspidatus* formed a distinct clade within Malacostraca, diverging from *Hyallela azteca* approximately 262 million years ago (MYA). Notably, the estimated divergence times between *C. bicuspidatus* and both *H. azteca* (316–397.7 MYA) and *Penaeus chinensis* (241–288.7 MYA) fall within overlapping 95% highest posterior density (HPD) intervals, which are consistent with estimates obtained from the TimeTree web server. This consistency further supports the robustness of the phylogenetic reconstruction and underscores the reliability of the underlying molecular dataset, which was derived from RNA-seq data. The transcriptome-based approach likely enhanced phylogenetic resolution by capturing a broad and functionally relevant repertoire of expressed genes.

**Table 1.** Orthogroup assignment and gene clustering statistics across 13 crustacean species.

Number of genes	250,870
Number of genes in orthogroups	217,500
Percentage of genes in orthogroups	86.7%
Number of orthogroups	20,741
Number of single-copy orthogroups	250

Gene family evolution analysis revealed that *C. bicuspidatus* experienced 28 gene family expansions and 152 contractions relative to its closest phylogenetic neighbors. While gene family contraction may suggest genomic streamlining, the expanded gene families are of particular interest as potential drivers of lineage-specific innovations. Gene gains are often associated with the acquisition of novel functions or enhanced adaptability to environmental pressures (Choi et al. 2025). Thus, investigating the functional significance of these expanded gene families could offer valuable insights into the ecological strategies and evolutionary trajectory of *C. bicuspidatus*.

**Figure 3.**
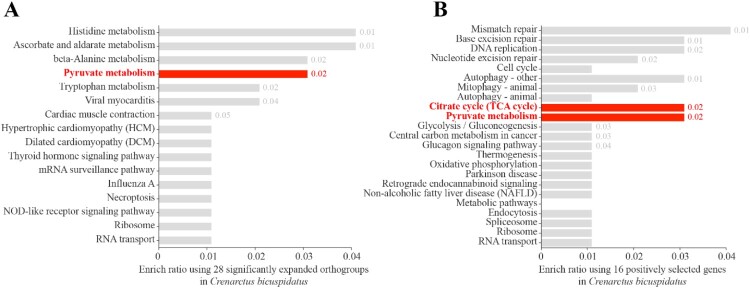
KEGG pathway enrichment analysis of gene sets from *Crenarctus bicuspidatus*. (A) Enriched KEGG pathways among 28 significantly expanded gene families identified using CAFE. (B) Enriched KEGG pathways among 16 positively selected genes identified using the branch-site model in CodeML. Pathway enrichment analysis was conducted using KOBAS. Pyruvate metabolism and the TCA cycle pathways are highlighted in red. The values displayed next to each bar indicate *p-*values; only statistically significant values (*p* < 0.05) are labeled.

We therefore examined the functions of the 28 orthogroups that were significantly expanded in *C. bicuspidatus* (Table 2). KEGG pathway enrichment analysis was conducted to explore the biological roles of these gene families (Figure 3A). The analysis revealed that the most enriched pathway was pyruvate metabolism, followed by several other metabolic and signaling pathways, including histidine metabolism, ascorbate and aldarate metabolism, beta-alanine metabolism, and tryptophan metabolism. The enrichment of pyruvate metabolism suggests that expansions in gene families related to energy metabolism may have played a critical role in the adaptive evolution of *C. bicuspidatus*. Pyruvate acts as a central node in cellular metabolism, linking glycolysis to the tricarboxylic acid (TCA) cycle and various biosynthetic processes (Vander Heiden et al. 2009). Among the 13 crustacean species examined, *C. bicuspidatus* is distinguished by its exceptionally long developmental period, extending far beyond that of the other taxa included in this study (Booth et al. 2005) (Table S1). This life-history trait may impose unique physiological demands across prolonged ontogenetic stages. In this context, the significantly expanded gene families identified in *C. bicuspidatus* could reflect molecular adjustments that support its extended developmental timeline. Although direct functional validation is required, this ecological feature provides a plausible evolutionary framework for interpreting the distinct genomic characteristics observed in this species. Therefore, the expansion of genes in this pathway may reflect enhanced metabolic flexibility or efficiency, potentially offering selective advantages under specific ecological or environmental conditions.

**Table 2.** Significantly expanded gene families in *C. bicuspidatus* and their functional annotations.

	Gene name	PFAM domains	Gene numbers of *C. bicuspidatus* in each orthogroups
OG0000029.fa	–	DDE_Tnp_4	14 (8%)
OG0000034.fa	znf711	NHL	42 (25%)
OG0000070.fa	MYH7	IQ, Myosin_N, Myosin_head, Myosin_tail_1	10 (8%)
OG0000093.fa	ZBTB7B	BTB	9 (9%)
OG0000167.fa	SERPINB1	Serpin	10 (14%)
OG0000228.fa	–	MADF_DNA_bdg	12 (21%)
OG0000257.fa	ATL2	GBP, GBP_C	3 (5%)
OG0000315.fa	prx	CH, KASH, Spectrin	22 (46%)
OG0000337.fa	UBC	ubiquitin	8 (17%)
OG0000357.fa	ZBED8	DUF4371, Dimer_Tnp_hAT	2 (4%)
OG0000409.fa	Tm2	Tropomyosin, Tropomyosin_1	14 (34%)
OG0000413.fa	–	–	8 (19%)
OG0000473.fa	sls	–	17 (44%)
OG0000476.fa	LDB3	DUF4749, PDZ, PDZ_2	8 (21%)
OG0000581.fa	tim	TIMELESS	3 (8%)
OG0000606.fa	CLK2	Pkinase	9 (27%)
OG0000607.fa	TNNI1	–	16 (48%)
OG0000615.fa	CPVL	Peptidase_S10	3 (9%)
OG0000703.fa	THOC5	FimP	2 (6%)
OG0000736.fa	PRRC2C	BAT2_N	12 (40%)
OG0000754.fa	FYCO1	FYVE, RUN	17 (56%)
OG0000813.fa	LDB3	DUF4749, LIM, PDZ	15 (51%)
OG0000896.fa	RNF31	–	13 (48%)
OG0000913.fa	PABPN1	–	2 (7%)
OG0000965.fa	STK11IP	LIP1, LRR_4, LRR_8	4 (15%)
OG0000978.fa	BIN3	THAP, Tnp_*P*_element, Tnp_*P*_element_C	2 (7%)
OG0001023.fa	TSC22D2	TSC22	7 (28%)
OG0004776.fa	ZNF784	–	6 (42%)

### 3.3. Molecular signatures of glycolytic adaptation in *C. bicuspidatus*

To investigate signatures of adaptive evolution, KEGG pathway enrichment analysis was performed on 16 genes under positive selection in *C. bicuspidatus*. This analysis revealed strong enrichment in two core metabolic pathways: pyruvate metabolism and the TCA cycle (Figure 3B). These pathways are central to cellular respiration, converting carbohydrates into ATP and biosynthetic intermediates. Their adaptive evolution may therefore reflect metabolic optimization in response to environmental pressures.

Interestingly, pyruvate metabolism was also enriched among significantly expanded orthogroups (Figure 3A), suggesting that this pathway has not only undergone gene family expansion but is also under positive selective pressure. This dual signal implies a central evolutionary role for the pyruvate metabolic pathway in *C. bicuspidatus*, potentially enhancing energy efficiency, metabolic flexibility, or stress responsiveness in dynamic coastal environments.

The downstream process, the TCA cycle, represents the key energy-generating hub of aerobic metabolism (Martínez-Reyes and Chandel 2020). The presence of positively selected genes within this highly conserved pathway suggests that even core metabolic routes are subject to evolutionary fine-tuning. Such changes may modulate ATP production, redox homeostasis, or metabolic flux in response to environmental variables such as temperature shifts, fluctuating oxygen levels, or nutrient availability, all of which are common features of shallow coastal habitats (O'Leary et al. 2019; Martínez-Reyes and Chandel 2020; Shahreen et al. 2025).

**Figure 4.**
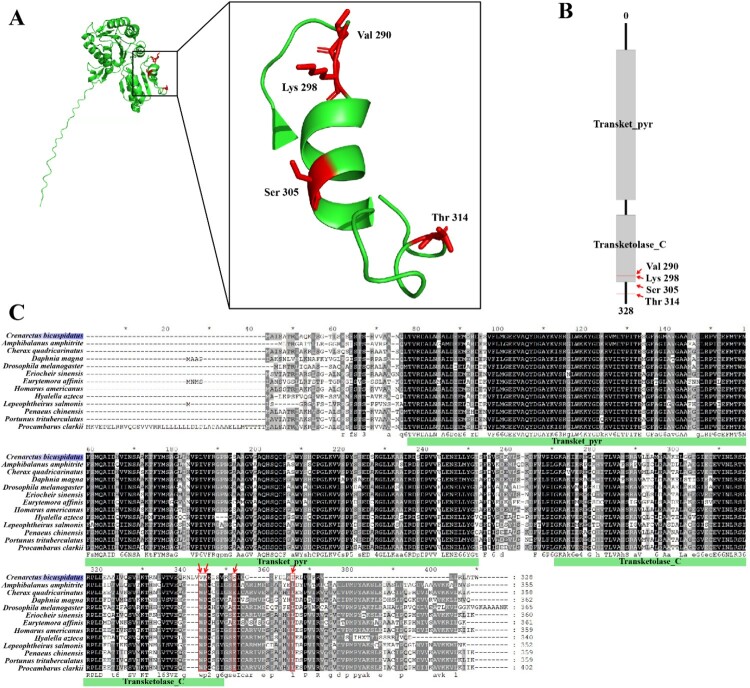
Positively selected sites in the pyruvate dehydrogenase (PDH) complex of *Crenarctus bicuspidatus*. (A) Three-dimensional structure of the PDH protein shown as a green ribbon diagram. Four positively selected amino acid residues (Val290, Lys298, Ser305, and Thr314), identified using the branch-site model, are highlighted in red. (B) Schematic diagram of the PDH protein domain architecture, including the *Transket_pyr* and *Transketolase_C* domains. Two positively selected residues (Val290 and Lys298) are located within the *Transketolase_C* domain and are marked with red arrows. (C) Multiple sequence alignment (MSA) of PDH orthologs across 13 Crustacean species. Conserved residues appear white on a black background; dashes indicate alignment gaps. Red arrows denote the positions of positively selected sites. Green bars below the alignment indicate domain boundaries corresponding to the *Transket_pyr* and *Transketolase_C* regions.

Since *C. bicuspidatus* exhibited the longest developmental duration among the examined species, such an extended ontogenetic period may impose distinct physiological and ecological demands, potentially shaping lineage-specific genomic features that support sustained growth and survival (Table S1). The larval development duration of *C. bicuspidatus* (150 days) was significantly longer than that of the other comparative taxa (mean ± SD = 39.8 ± 45.47 days). Statistical analysis identified *C. bicuspidatus* as a significant outlier within the dataset (Z-score = 2.42, *p* < 0.01), suggesting a distinct life-history strategy compared to the other 12 species. Therefore, *C. bicuspidatus* was designated as the foreground branch to identify genes exhibiting signatures of lineage-specific evolutionary pressure. In this analysis we detected 16 PSGs in *C. bicuspidatus*. And some PSGs exhibited extremely high ω value on the foreground branch, which corresponds to the upper computational limit of the program (Álvarez-Carretero et al. 2023). This often occurs when information is limited, such as in cases with very small dS values. Nevertheless, the identification of these genes as PSGs remains robust because it is based on the Likelihood Ratio Test (LRT). As these genes yielded statistically significant LRT result (*p* < 0.05), the evidence for positive selection is statistically supported regardless of the specific numeric value of the ω ratio. Among the 16 PSGs, we identified pyruvate dehydrogenase complex (PDH), a key mitochondrial enzyme (Figure 4A and Tables 3, Table S2)., Structural modeling and domain annotation revealed that two of the positively selected residues (Val290 and Lys298) are located within the conserved Transketolase_C domain of the PDH protein (Figure 4B). Multiple sequence alignment across 13 crustacean species confirmed that these residues are unique to *C. bicuspidatus*, indicating species-specific adaptive evolution (Figure 4C). Given the highly conserved function of the PDH complex and its central role in energy metabolism, these amino acid changes may influence enzymatic kinetics, regulatory interactions, or structural stability (Jiménez-Osés et al. 2014; Vanella et al. 2024; Anna Sajeevan et al. 2025). Together, the enrichment of energy-related pathways, particularly pyruvate metabolism and the TCA cycle, suggests that glycolytic adaptation has played a central role in the evolutionary history of *C. bicuspidatus*. The combination of gene family expansion and adaptive amino acid substitutions in key metabolic enzymes, such as PDH, indicates that natural selection has acted on both genomic structure and protein function to optimize energy metabolism. These multilayered adaptations likely reflect the prolonged energetic demands associated with an extended larval phase, where metabolic flexibility confers a selective advantage for surviving diverse environmental challenges during development. Collectively, our results point to coordinated evolutionary adjustments that fine-tune core metabolic processes and may contribute to the ecological success of *C. bicuspidatus* within its habitat. However, because the present analyses relied on a single individual for *de novo* assembly, the PSG signals identified here capture lineage-level evolutionary patterns rather than population-level selection processes. Although single-individual assemblies remain standard practice for generating high-quality reference genomes, they do not incorporate within-population genetic variation, which is essential for evaluating ongoing or recent selection. Future studies incorporating population-scale sampling and population genomics approaches will therefore be required to determine whether the PSGs identified in this study are also subject to selection within *C. bicuspidatus* populations.

**Table 3.** Statistical evidence for positive selection in *C. bicuspidatus* based on the branch-site model.

Gene name	Null model (np)	Alternative model (np)	LRTs (*p*-value)	Site class	0	1	2a	2b
*pyruvate dehydrogenase complex* (*PDH*)	–8095.200323 (27)	–8090.190643 (28)	0.002	Proportion	0.79	0.06	0.14	0.01
				Background ω	0.02	1	0.02	1
				Foreground ω	0.02	1	7.85	7.85
	Positively selected sites (based on BEB analysis)	2 Q (0.51), 40 S (0.952)*, 71 M (0.712), 83 M (0.74), 89 L (0.703), 126 L (0.56), 130 L (0.864), 135 S (0.903), 168 S (0.654), 179 S (0.913), 194 S (0.665), 200 K (0.733), 208 V (0.786), 235 M (0.83), 239 K (0.886), 242 G (0.863), 262 A (0.923), 263 A (0.807), 267 S (0.999)**, 272 R (0.707), 280 R (0.872), 281 V (0.999)**, 282 K (1)**, 283 E (0.887), 284 L (0.769), 285 S (0.868), 286 V (0.842), 287 R (0.910), 289 S (0.999)**, 292 G (0.863), 294 F (0.998)**, 295 D (0.996)**, 296 L (0.875), 297 W (0.999)**, 298 T (0.999)**, 299 R (0.999)**, 300 L (0.993)**, 302 L (0.858)						

Abbreviations: np, number of parameters; LRT, likelihood ratio test; BEB, Bayes Empirical Bayes.

## 5. Conclusion

This study provides the first comprehensive characterization of the mitochondrial and transcriptomic features of *C. bicuspidatus*, shedding light on its evolutionary history and metabolic adaptations. Analyses of gene family expansion and positive selection consistently pointed to energy-related pathways, particularly pyruvate metabolism and the TCA cycle. These findings suggest that glycolytic adaptation plays a critical role in supporting the exceptionally prolonged larval development of *C. bicuspidatus*, a distinct life-history trait confirmed as a significant statistical outlier in our comparative analysis. Our results underscore the importance of metabolic pathway evolution in shaping species-specific adaptive strategies among marine crustaceans. Future functional studies encompassing a broader range of slipper lobsters are warranted to further elucidate the genomic underpinnings of life-history and ecological diversification.

## Authors contributions

**Hyeongwoo Choi:** Conceptualization; Software; Formal analysis; Investigation; Data Curation; Writing – Original Draft; Writing – Review & Editing; Visualization. **Hyeongju Choi:** Data Curation; Investigation; Writing – Review & Editing. **Chungkug Park:** Writing – Review & Editing. **Won-Kyo Jung:** Writing – Review & Editing. **Tae-Hoon Kim**: Writing – Review & Editing. **Yun Keun An:** Writing – Review & Editing.

## Informed consent

All authors consent to the publication of this study.

## Supplementary Material

Supplemental Material

## Data Availability

The raw sequence data generated in this study is publicly available in the NCBI GenBank under accession number SRA (BioProject: PRJNA1250710). The complete mitochondrial genome of *C. bicuspidatus* is available in GenBank under accession number PV197094.
