# Supplementary material for: Comparative phylotranscriptomics reveals glycolytic adaptation associated with prolonged larval development in the coastal slipper lobster Crenarctus bicuspidatus
Source: Anim Cells Syst (Seoul). 2026 Mar 21;30(1):346–56. doi: 10.1080/19768354.2026.2643995 (PMC13007470; doi:10.1080/19768354.2026.2643995)
Supplement: Supplemental Material [file TACS_A_2643995_SM7711.docx]

[Article] Running Head: Glycolytic adaptations in *Crenarctus bicuspidatus*

**Title**

**Comparative phylotranscriptomics reveals glycolytic adaptation associated with prolonged larval development in the coastal slipper lobster Crenarctus bicuspidatus**

**Supplementary Table 1. Larval to adult developmental duration across 13 species. Note: The asterisk (*) indicates that C. bicuspidatus is a significant statistical outlier within this dataset (Z-score = 2.54, *p* < 0.01, calculated based on the mean and standard deviation).**

| **Species** | **Duration (days)** | **Reference** |
| --- | --- | --- |
| ***Crenarctus bicuspidatus*** | 150* | (Booth et al. 2005) |
| *Procambarus clarkii* | 25 | (Patoka et al. 2013) |
| *Cherax quadricarinatus* | 15 | (Rigg et al. 2021) |
| *Homarus americanus* | 99 | (Quinn et al. 2013) |
| *Penaeus chinensis* | 35 | (Park and Hur 2005) |
| *Eriocheir sinensis* | 14 | (Sui et al. 2011) |
| *Portunus trituberculatus* | 19 | (Pyen 1970) |
| *Hyalella azteca* | 98 | (March 1977) |
| *Amphibalanus amphitrite* | 17 | (Al-Aidaroos and Satheesh 2014) |
| *Eurytemora affinis* | 12 | (Karlsson et al. 2018) |
| *Lepeophtheirus salmonis* | 17.5 | (Whelan 2010) |
| *Daphnia magna* | 10 | (Thakur and Kocher) |
| *Drosophila melanogaster* | 6 | (Brischigliaro et al. 2023) |

**Supplementary Table 2. Statistical results of 16 positively selected genes using *C. bicuspidatus* as a foreground branch by branch-site model. Abbreviations: BEB, Bayes Empirical Bayes. Positively selected sites inferred by BEB with posterior probability > 0.95 (*p* < 0.05) are shown.**

| **Gene name** | **LRTs** | ***p*-value** | **Foreground dN/dS ratio** | **Aligned length (bp)** | **Positively selected sites (based on BEB analysis)** |
| --- | --- | --- | --- | --- | --- |
| *COX18* | 5.11 | 0.02 | 3.84 | 702 | 222 P (0.99), 224 I (0.99),226 R (0.99), 230 S (0.99), 233 K (0.98) |
| *PDH* | 10.02 | 0.001 | 7.85 | 915 | 40 S (0.95), 267 S (0.99), 281 V (0.99), 282 K (1), 289 S (0.99), 294 F (0.99), 295 D (0.99), 297 W (0.99), 298 T (0.99), 299 R (0.99), 300 L (0.99) |
| *PCNA* | 4.26 | 0.04 | 472.42 | 777 | 105 S (0.97), 123 I (0.97), 159 T (0.95) |
| *VTA1* | 9.6 | 0.002 | 1 | 489 | 6 L (0.98), 54 F (0.98) |
| *DPH1* | 7.28 | 0.007 | 1 | 1,053 | 280 E (0.95) |
| *MPHOSPH10* | 3.9 | 0.05 | 79.73 | 1,434 | 361 S (0.97) |
| *VPS25* | 4.17 | 0.04 | 1 | 471 | 65 T (0.99), 117 R (0.99) |
| *MRPL9* | 4.73 | 0.03 | 5.59 | 657 | 198 V (0.99) |
| *RMND5A* | 9.44 | 0.002 | 1 | 1,140 | - |
| *EIF3B* | 4.68 | 0.03 | 20.38 | 2,016 | 84 V (0.96), 99 A (0.99), 143 G (0.96) |
| *ATG9A* | 15.1 | 0.0 | 1 | 1,962 | - |
| *CTNNBL1* | 15.9 | 0.0 | 999 | 1,263 | 183 V (0.98) |
| *WDR77* | 6.88 | 0.0 | 26.25 | 588 | 23 S (0.96) |
| *F8A1* | 7.08 | 0.008 | 510.97 | 702 | 195 S (0.99) |
| *TMEM185A* | 7.1 | 0.008 | 4.3 | 552 | 162 S (0.96) |
| *NDUFA6* | 6.09 | 0.01 | 16.43 | 366 | 4 S (0.99) |

**References**

Al-Aidaroos AM, Satheesh S. 2014. Larval development and settlement of the barnacle *Amphibalanus amphitrite* from the Red Sea: Influence of the nauplii hatching season. Oceanol Hydrobiol Stud. 2014/06/01;43:170-177.

Booth JD, Webber WR, Sekiguchi H, Coutures E. 2005. Diverse larval recruitment strategies within the Scyllaridae. N Z J Mar Freshwat Res.39:581-592.

Brischigliaro M, Fernandez-Vizarra E, Viscomi C. 2023. Mitochondrial neurodegeneration: lessons from *Drosophila melanogaster* models. Biomolecules.13:378.

Karlsson K, Puiac S, Winder M. 2018. Life-history responses to changing temperature and salinity of the Baltic Sea copepod *Eurytemora affinis*. Mar Biol. 2018/01/18;165:30.

March BGEd. 1977. The effects of photoperiod and temperature on the induction and termination of reproductive resting stage in the freshwater amphipod *Hyalella azteca* (Saussure). Can J Zool.55:1595-1600.

Park IS, Hur JW. 2005. Early larval growth of flesh prawn (*Fenneropenaeus chinensis*). Korean J Environ Biol.23:27-31.

Patoka J, PETRTÝL M, KALOUS L. 2013. Growth of juvenile red swamp crayfish (*Procambarus clarkii*)(Decapoda: Cambaridae) reared in groups consisting of either sibling and non sibling individuals. Acta Soc Zool Bohem.77:67-71.

Pyen CK. 1970. Propagation of the blue crab, *Portunus trituberculatus* (Miers). Korean Journal of Fisheries and Aquatic Sciences.3:187-198.

Quinn BK, Rochette R, Ouellet P, Sainte-Marie B. 2013. Effect of temperature on dDevelopment rate of larvae from Cold-Water American Lobster (*Homarus Americanus*). J Crust Biol.33:527-536.

Rigg DP, Courtney RL, Jones CM, Seymour JE. 2021. Morphology and weight-length relationships for the first six instars of *Cherax quadricarinatus* (von Martens, 1868). Freshw Crayfish.26:9-16.

Sui L, Wille M, Cheng Y, Wu X, Sorgeloos P. 2011. Larviculture techniques of Chinese mitten crab *Eriocheir sinensis*. Aquac.315:16-19.

Thakur A, Kocher D. Laboratory studies on developmental stages and life cycle of *Daphnia magna*. International Journal of Fauna and Biological Studies.5:4-8.

Whelan K. 2010. A review of the impacts of the salmon louse, *Lepeophtheirus salmonis* (Krøyer, 1837) on wild salmonids. AST.1-27.
